# Supplementary material for: End Sequence Analysis Toolkit (ESAT) expands the extractable information from single-cell RNA-seq data
Source: Genome Res. 2016 Oct;26(10):1397–410. doi: 10.1101/gr.207902.116 (PMC5052061; doi:10.1101/gr.207902.116)
Supplement: Supplemental Material [file supp_gr.207902.116_Supp_Materials.tar.gz › ESAT-esat_v0.1/doc/html/classumms_1_1core_1_1annotation_1_1_annotation_file_reader.html]

ESAT: umms.core.annotation.AnnotationFileReader Class Reference


ESAT


- Main Page
- Related Pages
- Packages
- Classes
- Files

- Class List
- Class Index
- Class Hierarchy
- Class Members

All Classes Namespaces Files Functions Variables Enumerator Pages

- umms
- core
- annotation
- AnnotationFileReader

Classes |
Static Public Member Functions |
List of all members

umms.core.annotation.AnnotationFileReader Class Reference

|  |  |
| --- | --- |
| Static Public Member Functions | |
| static< TextendsAnnotation >  AnnotationList< T > | load (File file, Class< T > clazz, TabbedReader.Factory<?extends T > factory) throws IOException |
|  | |
| static< TextendsAnnotation >  AnnotationList< T > | load (File file, Class< T > clazz, TabbedReader.Factory<?extends T > factory, CoordinateSpace cs) throws IOException |
|  | |
| static< TextendsAnnotation >  AnnotationList< T > | load (File file, Class< T > clazz, TabbedReader.Factory<?extends T > factory, CoordinateSpace cs, Collection< Predicate<?super T >> filters) throws IOException |
|  | |
| static< TextendsAnnotation >  AnnotationList< T > | load (File file, Class< T > clazz, TabbedReader.Factory<?extends T > factory, CoordinateSpace cs, Predicate<?super T > filter) throws IOException |
|  | |
| static< TextendsAnnotation >  CloseableIterator< T > | read (File file, Class< T > clazz, TabbedReader.Factory<?extends T > factory, Predicate<?super T > filter) throws IOException |
|  | |
| static< TextendsAnnotation >  CloseableIterator< T > | read (File file, Class< T > clazz, TabbedReader.Factory<?extends T > factory) throws IOException |
|  | |

## Detailed Description

Author
:   engreitz This class is responsible for reading annotation files and generating AnnotationLists from them. Parsing logic is partially copied from broad.core.annotation.AnnotationReader

## Member Function Documentation

|  |  |  |  |
| --- | --- | --- | --- |
| static <TextendsAnnotation> AnnotationList<T> umms.core.annotation.AnnotationFileReader.load | ( | File | *file*, |
|  |  | Class< T > | *clazz*, |
|  |  | TabbedReader.Factory<?extends T > | *factory* |
|  | ) |  | throws IOException |

static

Here is the caller graph for this function:

|  |  |  |  |
| --- | --- | --- | --- |
| static <TextendsAnnotation> AnnotationList<T> umms.core.annotation.AnnotationFileReader.load | ( | File | *file*, |
|  |  | Class< T > | *clazz*, |
|  |  | TabbedReader.Factory<?extends T > | *factory*, |
|  |  | CoordinateSpace | *cs* |
|  | ) |  | throws IOException |

static

Here is the call graph for this function:

|  |  |  |  |
| --- | --- | --- | --- |
| static <TextendsAnnotation> AnnotationList<T> umms.core.annotation.AnnotationFileReader.load | ( | File | *file*, |
|  |  | Class< T > | *clazz*, |
|  |  | TabbedReader.Factory<?extends T > | *factory*, |
|  |  | CoordinateSpace | *cs*, |
|  |  | Collection< Predicate<?super T >> | *filters* |
|  | ) |  | throws IOException |

static

Here is the call graph for this function:

|  |  |  |  |
| --- | --- | --- | --- |
| static <TextendsAnnotation> AnnotationList<T> umms.core.annotation.AnnotationFileReader.load | ( | File | *file*, |
|  |  | Class< T > | *clazz*, |
|  |  | TabbedReader.Factory<?extends T > | *factory*, |
|  |  | CoordinateSpace | *cs*, |
|  |  | Predicate<?super T > | *filter* |
|  | ) |  | throws IOException |

static

Reads an Annotation file line by line and return an AnnotationList containing the results

Parameters
:   |  |  |
    | --- | --- |
    | <T> |  |
    | file | File containing annotations |
    | clazz | The class of the desired AnnotationList |
    | cs | CoordinateSpace with which to initialize the AnnotationList. Can be null. |
    | factory | Factory for parsing and creating the annotation type |
    | filter | Annotation filters to control the subset of annotations that are stored in the AnnotationList. |

Returns
:   AnnotationList containing all annotations from file that pass the filter

Exceptions
:   |  |  |
    | --- | --- |
    | IOException |  |

Here is the call graph for this function:

|  |  |  |  |
| --- | --- | --- | --- |
| static <TextendsAnnotation> CloseableIterator<T> umms.core.annotation.AnnotationFileReader.read | ( | File | *file*, |
|  |  | Class< T > | *clazz*, |
|  |  | TabbedReader.Factory<?extends T > | *factory*, |
|  |  | Predicate<?super T > | *filter* |
|  | ) |  | throws IOException |

static

Read an Annotation file line by line in a buffered format and return an iterator over the results. Should be refactored with the "load" function as appropriate ("load" can call this function and then add it to its Annotation list)

Parameters
:   |  |  |
    | --- | --- |
    | file |  |
    | clazz |  |
    | factory |  |
    | cs |  |
    | filter |  |

Returns

Here is the caller graph for this function:

|  |  |  |  |
| --- | --- | --- | --- |
| static <TextendsAnnotation> CloseableIterator<T> umms.core.annotation.AnnotationFileReader.read | ( | File | *file*, |
|  |  | Class< T > | *clazz*, |
|  |  | TabbedReader.Factory<?extends T > | *factory* |
|  | ) |  | throws IOException |

static

Here is the call graph for this function:

---

The documentation for this class was generated from the following file:

- java/umms/core/annotation/AnnotationFileReader.java


---

Generated on Fri Aug 15 2014 15:11:08 for ESAT by  

 1.8.7
